# Cellular Levels of Oxidative Stress Affect the Response of Cervical Cancer Cells to Chemotherapeutic Agents

**DOI:** 10.1155/2014/574659

**Published:** 2014-11-16

**Authors:** Maria Filippova, Valery Filippov, Vonetta M. Williams, Kangling Zhang, Anatolii Kokoza, Svetlana Bashkirova, Penelope Duerksen-Hughes

**Affiliations:** ^1^Department of Basic Sciences, Loma Linda University School of Medicine, 11021 Campus Street, 101 Alumni Hall, Loma Linda, CA 92354, USA; ^2^Department of Pharmacology and Toxicology, University of Texas Medical Branch, Galveston, TX 77555, USA

## Abstract

Treatment of advanced and relapsed cervical cancer is frequently ineffective, due in large part to chemoresistance. To examine the pathways responsible, we employed the cervical carcinoma-derived SiHa and CaSki cells as cellular models of resistance and sensitivity, respectively, to treatment with chemotherapeutic agents, doxorubicin, and cisplatin. We compared the proteomic profiles of SiHa and CaSki cells and identified pathways with the potential to contribute to the differential response. We then extended these findings by comparing the expression level of genes involved in reactive oxygen species (ROS) metabolism through the use of a RT-PCR array. The analyses demonstrated that the resistant SiHa cells expressed higher levels of antioxidant enzymes. Decreasing or increasing oxidative stress led to protection or sensitization, respectively, in both cell lines, supporting the idea that cellular levels of oxidative stress affect responsiveness to treatment. Interestingly, doxorubicin and cisplatin induced different profiles of ROS, and these differences appear to contribute to the sensitivity to treatment displayed by cervical cancer cells. Overall, our findings demonstrate that cervical cancer cells display variable profiles with respect to their redox-generating and -adaptive systems, and that these different profiles have the potential to contribute to their responses to treatments with chemotherapy.

## 1. Introduction

Worldwide, cervical cancer is the second most common cancer in women; approximately 400 000 new cases of this disease are diagnosed each year, of which approximately half will lead to death. The causative agents of most cases of cervical carcinomas are the high-risk types of human papillomaviruses (HPV). When cervical carcinomas are detected at early or preinvasive stages, they are often curable with local treatments, most of which are based on ablative approaches. Unfortunately, a significant proportion of patients diagnosed with invasive cervical cancer suffer relapses following initial treatment. For this reason, the development of novel and effective therapeutic interventions, such as those based on molecular techniques, remains an important priority [1, 2].

More than 20 different chemotherapeutic agents are now considered active in the treatment of cervical carcinomas, in that they produce response rates of 15%–20%. Recent and ongoing trials are also likely to identify additional active drugs [3]. The low response rate to these agents can be attributed to the fact that invasive cervical cancer appears to be relatively chemoresistant, as compared to other gynecologic tumors such as those of the breast or ovaries [3]. Studies in breast, prostate, and colorectal cancers have shown that many factors can contribute to chemoresistance, including an individual's genetic background as well as epigenetic factors [4]. However, such studies have not yet analyzed the causes of chemoresistance in cervical cancer. An understanding of the molecular events that lead to chemoresistance in the cells comprising cervical carcinomas may lead to the discovery of new targets for chemical intervention.

CaSki and SiHa cells represent useful cellular models for cervical carcinoma, as both lines contain an integrated form of HPV16. Interestingly, however, they respond quite differently to treatment with agents that induce cell death through both intrinsic [5, 6] and extrinsic [7] apoptotic pathways. In spite of the significant differences in the molecular pathways involved (e.g., DNA-damaging agents* versus* ligands that induce ligand-mediated apoptosis), one common observation stands out: CaSki cells are more sensitive to each of these treatments than are SiHa cells. The reason(s) for these dramatically different responses have not yet been identified. It has been suggested that differences in the levels of p53 [8, 9] and/or procaspase 8 [7, 10] could contribute.

In the current study, we compared the proteomic profiles of SiHa and CaSki cells and identified pathways with the potential to contribute to the differential response to chemotherapeutic agents. We then extended these findings by analyzing and comparing the expression level of genes involved in reactive oxygen species (ROS) metabolism through the use of an RT-PCR array. Both sets of analyses demonstrated that the resistant SiHa cells expressed higher levels of antioxidant enzymes. Decreasing or increasing oxidative stress using pharmacological agents led to protection or sensitization, respectively, in both cell lines, supporting the idea that cellular levels of oxidative stress affect responsiveness to treatment. Interestingly, the two agents tested, doxorubicin (DOX) and cisplatin, induced different profiles of ROS, and these differences appear to contribute to the differential sensitivity observed.

## 2. Materials and Methods

### 2.1. Reagents

Monoclonal *α*-NQO1, *α*-OXR1, and *α*-*β*-actin were obtained from Sigma-Aldrich, monoclonal *α*-SOD1, and *α*-GPX 1/2 from Santa Cruz Biotechnology, monoclonal *α*-SOD2 from BD Biosciences, and monoclonal *α*-PARP1 (Ab-2) from Millipore Corporation (Calbiochem).* tert*-Butyl hydroperoxide solution (tBHP),* N*-Acetyl-L-cysteine (NAC),* cis*-diammineplatinum(II) dichloride (cisplatin), doxorubicin hydrochloride (DOX), and DL-buthionine-(S-R)-sulfoximine (BSO) were purchased from Sigma-Aldrich.

### 2.2. Cell Culture

CaSki, SiHa, HeLa, and C33A cells (derived from human cervical carcinomas) were obtained from the ATCC (Manassas VA). All cells were cultured in modified Eagle medium (MEM) (CellGro). The medium was supplemented with 10% fetal bovine serum (Life Biosciences) and with penicillin (100 *μ*g/mL) and streptomycin (100 *μ*g/mL) (Sigma-Aldrich).

### 2.3. Cell Treatments and Cell Viability Assay

Cells (1 or 1.5 × 10^4^ cells per well) were seeded onto a 96-well plate and allowed to incubate for 24 h, after which they were treated with the indicated concentrations of drugs or tBHP. NAC or BSO was also added where indicated. Cell viability was monitored using crystal violet staining. The absorbance of each well was determined at 590 nm using a plate reader.

### 2.4. Proteomic Analysis

SiHa and CaSki cells (10^7^) were lysed in RIPA lysis buffer (Sigma-Aldrich) and sonicated. Cleared lysates were denatured, reduced, and alkylated as recommended by the TMT Mass Tagging Kit (Thermo Fisher Scientific) protocol. Trypsin was added at a protein/enzyme ratio of 30 : 1 by mass and the digestion was performed at 37°C overnight. Peptides were labeled with TMT (tandem-mass-tagging) reagents according to the manufacturer's protocol in duplicate and equal amounts of labeled peptides were combined to obtain one sample, which was separated into 9 fractions by strong cation exchange chromatography using TopTip columns (PolyLC). Elution was performed with increasing concentrations of KCl (from 0 to 0.5 M). Eluates were dried using a SpeedVac and then desalted using C18/hypercarb TopTip columns (PolyLC). Samples from each fraction were run in triplicate on an Orbitrap Pro mass spectrometer that was coupled to a nanoLC (Thermo Fisher Scientific), and the spectra obtained were analyzed with Proteome Discoverer 1.3 software against the Human International Protein Index (IPI) database. Peptides were identified with a FDR (false discovery rate) of less than 1%. Proteins were considered differentially expressed if the fold ratio was more than 1.5. Protein data were further analyzed using Ingenuity Pathway Analysis (IPA) software to identify differences in pathways and networks between cell lines.

### 2.5. Measurement of ROS in Cells by Flow Cytometry

Intracellular generation of hydrogen peroxide (H_2_O_2_), hydroxyl and peroxyl radicals (DCFDA), and superoxide (O_2_^−^) (DHE) was estimated using either the 5-(and-6)-carboxy-2′,7′-dichlorodihydrofluorescein diacetate (DCFDA) or dihydroethidium (DHE) membrane permeable probes (Life Biosciences). Reagents were diluted into culture media and then added to cells to a final concentration of 10 *μ*M. After treatment, the cells were collected in 1x PBS and analyzed using the Becton-Dickinson FACSCalibur flow cytometer (Becton-Dickinson, San Francisco, CA). DCFDA was detected in the FL-1 channel, while DHE was detected in the FL-2 channel. Data was collected in log scale and analyzed using Flow-Jo software.

### 2.6. Microscopy

CaSki and SiHa cells were seeded onto CultureSlides (Falcon) one day prior to treatment with DOX or cisplatin. The following day, cells were stained with DCFDA and DHE as described above (measurement of ROS in cells by flow cytometry). Fluorescent images were recorded using a Biorevo microscope (Keyence) at the same magnifications and the same settings.

### 2.7. Immunoblot Assays

For immunoblot analysis, 10^6^ cells were lysed in 100 *μ*L of Laemmli lysis buffer and lysates were sonicated and separated by SDS-PAGE. After transfer of protein onto Immobilon P membranes (Millipore Corporation) and blocking of the membrane with 1% bovine serum albumin dissolved in TBST, primary antibodies were applied overnight. Secondary ImmunoPure antibody (*α*-mouse or *α*-rabbit), conjugated with horseradish peroxidase (Thermo Fisher Scientific), was applied onto the membrane for 1 h and the detection of signal was performed using the chemiluminescent SuperSignal West Dura or Pico maximum-sensitivity substrate (Thermo Fisher Scientific).

### 2.8. RNA Isolation, RT-PCR, and qRT-PCR

Cells were plated onto a 10 cm tissue culture plate and allowed to grow to semiconfluency. RNA was isolated using Tri Reagent according to the manufacturer's protocol (Sigma-Aldrich). cDNA was synthesized using ImPromII reverse transcriptase (Promega) and an oligo(dT) primer. Primers for the 5′ and 3′ ends of the indicated genes were used to amplify PCR products.

Quantitative qRT-PCR was conducted using the Absolute QPCR Sybr green kit according to the manufacturer's protocol (ABgene). The observed gene concentrations were normalized using* PGK1* expression levels.

### 2.9. Oxidative Stress and Antioxidant Defense PCR Array

The PCR Microarray was performed according to the manufacturer's instructions (SABiosciences, a QIAGEN company, Valencia CA). Gene expression was compared according to the *C*_*T*_ value. Normalization was performed for each cDNA sample using the average of five housekeeping genes provided by manufacture.

### 2.10. Statistics

All assays were repeated at least three times and the results reported as mean ± standard deviation. Differences were analyzed by the Student's *t*-test. *P* ≤ 0.05 was regarded as significant.

## 3. Results

### 3.1. SiHa Cells Are More Resistant Than CaSki Cells to Doxorubicin- and Cisplatin-Induced Cell Death

**Figure 1:**
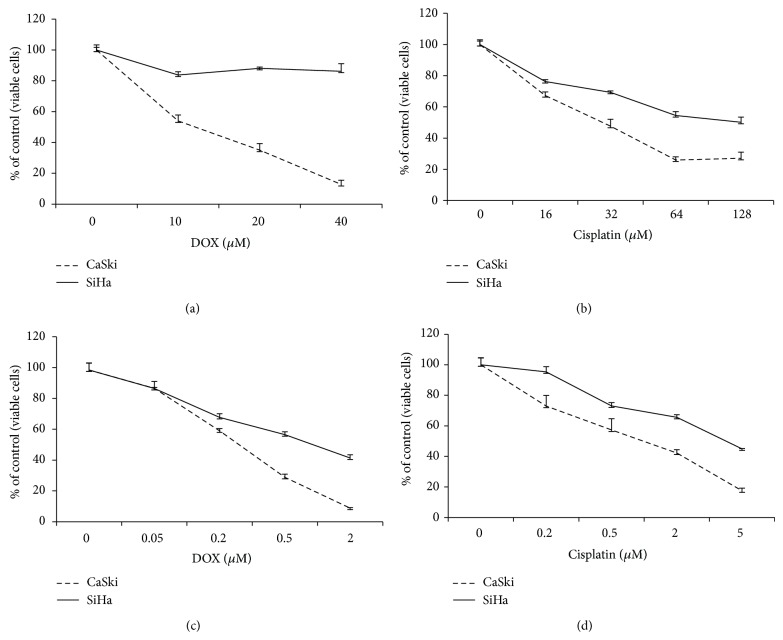
SiHa cells are more resistant than CaSki cells to treatment with the chemotherapeutic drugs DOX (a and c) and cisplatin (b and d). (a and b) SiHa and CaSki cells (1.5 × 10^4^ cells per well) were seeded into a 96-well plate, allowed to incubate overnight, and then treated with the indicated concentrations of drugs for 20 h. (c and d) SiHa and CaSki cells (0.5 × 10^4^ cells per well) were seeded into a 96-well plate, allowed to incubate overnight, and then treated with the indicated concentrations of drugs for 72 h. Viability was measured by crystal violet and the viability of untreated cells was set at 100%. Each measurement was done in triplicate and error bars indicate the standard deviations of the means.

Doxorubicin (DOX) and cisplatin are chemotherapeutic agents used to treat solid tumors, including cervical carcinomas [3]. To evaluate and compare the sensitivity of CaSki and SiHa cells to these chemotherapeutic drugs, cells were treated with increasing concentrations of DOX and cisplatin. For the initial set of experiments, relatively high concentrations were applied (10–40 *μ*M for DOX and 16–128 *μ*M for cisplatin) and crystal violet staining was used to monitor cell viability following treatment for 20 h (Figures 1(a) and 1(b)). With both treatments, we found that SiHa cells were more resistant to treatment than were the CaSki cells. For example, cisplatin at a concentration of ~30 *μ*M killed 50% of CaSki cells, while a loss of 50% viability for SiHa cells was observed at 128 *μ*M cisplatin (Figure 1(b)). Similar results were observed for DOX (Figure 1(a)). These experiments were then repeated using lower, more physiologically relevant concentrations (0.05 *μ*M to 2 *μ*M for DOX and 0.2 *μ*M to 5 *μ*M for cisplatin) [11], for a longer period of time (72 h) (Figures 1(c) and 1(d)). Again, we found that SiHa cells were more resistant to treatment than were CaSki cells.

### 3.2. Proteomic Analysis Identified Differences in Pathways Connected to p53 Activation, Mitochondrial Function, and Oxidative Stress

To identify differences in the pathways through which sensitive CaSki cells and the more resistant SiHa cells responded to drug treatment, we performed a comparative proteomic analysis. Identification and quantification of proteins were done by simultaneously running TMT-labeled trypsinized CaSki and SiHa lysates through an LTQ-Orbitrap mass spectrometer. The total number of proteins in which the level of expression between SiHa and CaSki cells differed by more than 1.5-fold was 430 (Table 1; see Supplementary Material available online at http://dx.doi.org/10.1155/2014/574659) and the detected range of differences in protein levels between these cells ranged from −6.0 to 6.9 fold. Seventy-six of these proteins were found to be upregulated, while the remainder was downregulated in SiHa cells as compared to CaSki cells.

**Figure 2:**
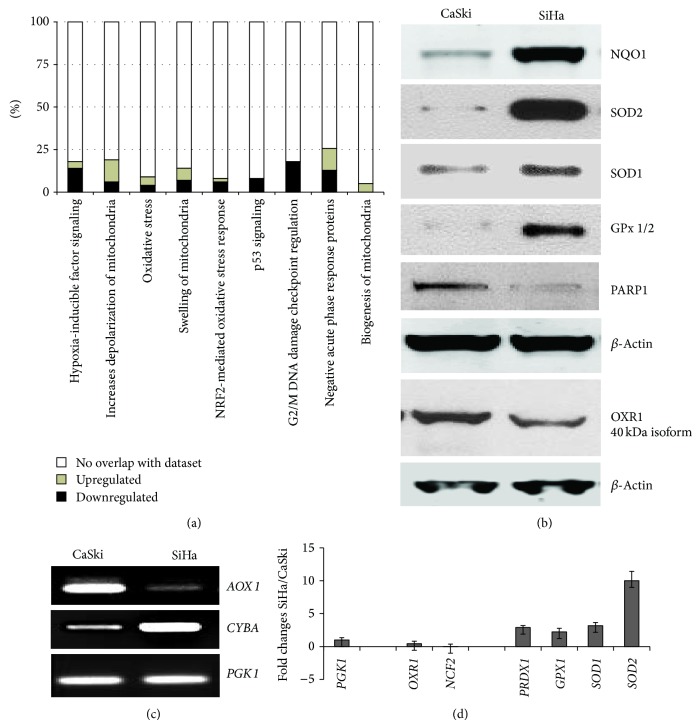
SiHa and CaSki cells differ in expression levels of proteins involved in the regulation of ROS. (a) Differential regulation of nine pathways in SiHa and CaSki cells. The percentage of genes downregulated in SiHa as compared to CaSki cells is shown in black, upregulated in grey. (b) Immunoblot analysis confirms differential expression. Lysates prepared from 10^6^ CaSki and SiHa cells using Laemmli lysis buffer were subjected to SDS-PAGE. Immunoblots were performed using antibodies directed against the indicated proteins. Loading was normalized by blotting for *β*-actin. (c and d) Transcription levels of genes related to oxidative stress as determined by semiquantitative RT-PCR (c) and by quantitative qRT-PCR (d) differ between SiHa and CaSki cells. Total RNA was isolated from 10^6^ cells of each cell line using Trizol Reagent (Sigma Aldrich) and cDNA was synthetized using oligo-dT. PCR and qPCR were performed using specific primers for the genes of interest. The PCR and qPCR products obtained using primers for the* PGK1* transcript were used to normalize to cDNA input. (d) Differences in gene expression are presented as fold changes (SiHa* versus* CaSki). Each measurement was done in triplicate and error bars indicate the standard deviations of the means.

To gain insight into the functions of these differentially expressed proteins, we employed the online IPA analysis (Ingenuity Systems) tool to group them into functionally related networks and pathways.  Figure 2(a) summarizes the 9 functions for which protein expression differs most between these two lines. Though HPV16 E6 accelerates the degradation of p53 [12], thereby significantly lowering its cellular level, our mass spectroscopy-based method was able to detect and quantify p53 in both SiHa and CaSki cells, demonstrating a 2.5-fold higher level of p53 in CaSki than in SiHa cells (Table 1, Supplementary Material). IPA analysis revealed that additional proteins within the p53 signaling pathways were downregulated in SiHa cells as compared to CaSki (Figure 2(a)). One downstream consequence of these differences in p53-linked pathways is the difference in the expression of proteins involved in G2/M DNA damage checkpoint regulation (Figure 2(a) and Table 1, Supplementary Material).

Some of the more remarkable differences in protein levels between SiHa and CaSki cells were detected in proteins involved in mitochondrial functions such as mitochondrial depolarization, swelling of mitochondria, and the biogenesis of mitochondria (Figure 2(a)). One important outcome of proper mitochondrial functioning involves the production and safe transport of radicals, as well as the maintenance of free radical homeostasis in the cell. Another group of pathways differentially activated between these cell lines is connected to DNA repair and the DNA damage response (Figure 2(a) and Table 1, Supplementary Material). Differences in the expression of proteins involved in mitochondrial status and DNA repair were accompanied by changes in the levels of proteins involved in the regulation of ROS levels (Figure 2(a)). For example, NAD(P)H dehydrogenase, quinine 1 (NQO1), peroxiredoxin 2 (PRDX2), and superoxide dismutase 1 (SOD1), which are responsible for inactivation of superoxide radicals, were found in higher levels in SiHa than in CaSki cells (Table 1, Supplementary Material).

To verify this proteomic data as well as the differences between these two cell lines with regards to expression of proteins involved in ROS metabolism, we evaluated the expression levels of a subset of the proteins involved in antioxidant defense by immunoblot (Figure 2(b)). Consistent with our proteomic data, the immunoblot analysis confirmed higher levels of NQO1 and SOD1 in SiHa cells and added glutathione peroxidase 1/2 (Gpx1/2) and SOD2 to the list of differentially-expressed genes (Figure 2(b)). A marker of DNA damage caused by oxidative stress, PARP1 was also detected at higher level in CaSki than in SiHa cells as assessed both by proteomic (Table 1, Supplementary Material) and immunoblot analyses (Figure 2(b)).

To further identify ROS-related genes with differential expression between SiHa and CaSki cells, we employed the Oxidative Stress and Antioxidant Defense PCR Array (SA Biosciences), which profiles the expression of 84 genes related to oxidative stress. We found that several of these genes were upregulated in SiHa (as compared to CaSki) cells and that a few were downregulated (Table 1). Genes whose expression was consistently downregulated in SiHa (relative to CaSki) cells included aldehyde oxidase 1 (*AOX1*) (Figure 2(c)), NADPH oxidase complex (*NCF2*) (Figure 2(d)), and oxidation resistance protein (*OXR1*) (Figure 2(d)); products of these genes are responsible for the production of reactive oxygen radicals. A reduced level of* OXR1* expression in SiHa cells as compared to CaSki cells was also confirmed by immunoblot (Figure 2(b)). Several genes that were upregulated in SiHa (Table 1) participate in scavenging radicals in one way or another. For example, cytochrome b-245, alpha polypeptide (*CYBA*) is a component of mitochondrial Complex III, which is involved in the transfer of electrons to Complex IV so that water can be formed (Figure 2(c) and Table 1). Other genes belong to various antioxidant systems. For example, SODs, GPXs, and PRDXs catalyze reactions that inactivate superoxide radicals (SODs) or H_2_O_2_ (GPXs and PRDXs). Overall, our immunoblot (Figure 2(b)), semiquantitative RT-PCR (Figure 2(c)), and quantitative qRT-PCR (Figure 2(d)) confirmed our initial results from the PCR array profiles. In summary, our data demonstrated significant differences between SiHa and CaSki cells with regards to the expression levels of genes and proteins involved in ROS metabolism and homeostasis.

### 3.3. Levels of ROS and Oxidative Stress-Induced Cell Death Are Higher in CaSki Than in SiHa Cells

**Figure 3:**
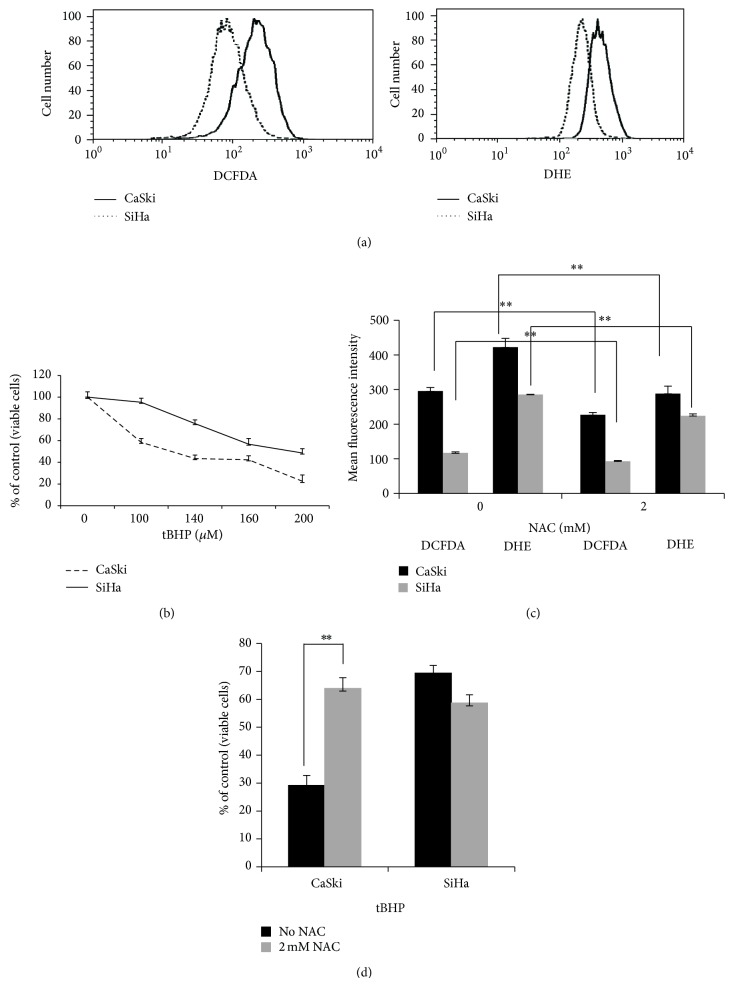
CaSki cells display higher levels of ROS than do SiHa cells (a), the cell viability of CaSki cells decreases more than in SiHa cells after treatment with tBHP (b). Pretreatment with NAC decreases ROS in CaSki and SiHa cells (c) and pretreatment with NAC protects cells from death induced by treatment with tBHP (d). (a) 10^5^ cells of each line were treated with 10 *μ*M DCFDA or 10 *μ*M DHE in media and then incubated in the dark at 37°C for 30 minutes. The cells were washed and, after trypsinization, were resuspended in 1x PBS and analysed by flow cytometry. A total of 10 000 events were measured per sample. (b) SiHa and CaSki cells (1.5 × 10^4^ cells per well) were seeded into a 96-well plate, allowed to incubate overnight, and then treated with the indicated concentrations of tBHP for 20 h. Viability was measured by crystal violet. The viability of untreated cells was set at 100%. Each measurement was done in triplicate and error bars indicate the standard deviations of the means. (c and d) Pretreatments with NAC were begun 24 h prior to treatment with tBHP (d). (c) The measurement of ROS levels was performed as described in (a) and (d) the measurements of cell viability were performed as described in (b).

The differential expression of pro- and antioxidant enzymes in CaSki and SiHa cells is expected to influence the baseline levels of ROS in these cells. To test this idea, SiHa and CaSki cells were stained with the DCFDA and DHE fluorescent dyes and the intensity of staining monitored by flow cytometry. Once DCFDA enters a cell, it is deacetylated by cellular esterases to form a nonfluorescent compound. ROS radicals such as H_2_O_2_, hydroxyl, and peroxyl radicals then oxidize this nonfluorescent substrate into 2′,7′-dichlorofluorescein, which is a highly fluorescent compound with maximum excitation and emission spectra of 495 nm and 529 nm, respectively [13]. In the case of DHE, its oxidation by superoxide results in hydroxylation at the 2-position. 2-hydroxyethidium exhibits a fluorescence excitation peak at ~400 nm [14]. Data presented in Figure 3(a) clearly demonstrates that SiHa cells display lower levels of the reactive oxygen species detected by both DCFDA and DHE than do CaSki cells.

These data suggested that cells with an elevated level of oxidative stress should be more susceptible to treatment with agents that further increase oxidative stress than cells with intrinsically lower levels of oxidative stress. To test this idea, we compared the cellular response to additional external oxidative stress by treating cells with tBHP for 20 h and then estimating the level of cell death. We found that CaSki cells, with their higher baseline levels of oxidative stress, were more susceptible to cell death caused by additional oxidative stress than were SiHa cells (Figure 3(b)).

We then exposed CaSki and SiHa cells to an antioxidant, NAC, prior to treatment with t-BHP. Cellular levels of ROS before and after pretreatment with NAC, as estimated by flow cytometry, are shown in Figure 3(c) and demonstrate that exposure to NAC decreased ROS levels as detected by DCFDA and DHE in both cell lines. Importantly, pretreatment with NAC increased the viability of the tBHP-sensitive CaSki cells. Pretreatment of SiHa cells with the same NAC concentration (140 *μ*M) did not affect their viability, presumably because they were already tBHP-resistant (Figure 3(d)).

Together, the data presented above suggest that internal levels of oxidative stress may affect cellular sensitivity to cytotoxic agents.

### 3.4. Changes in Cellular Oxidative Stress Affect the Response of Cells to Chemotherapeutic Agents

**Figure 4:**
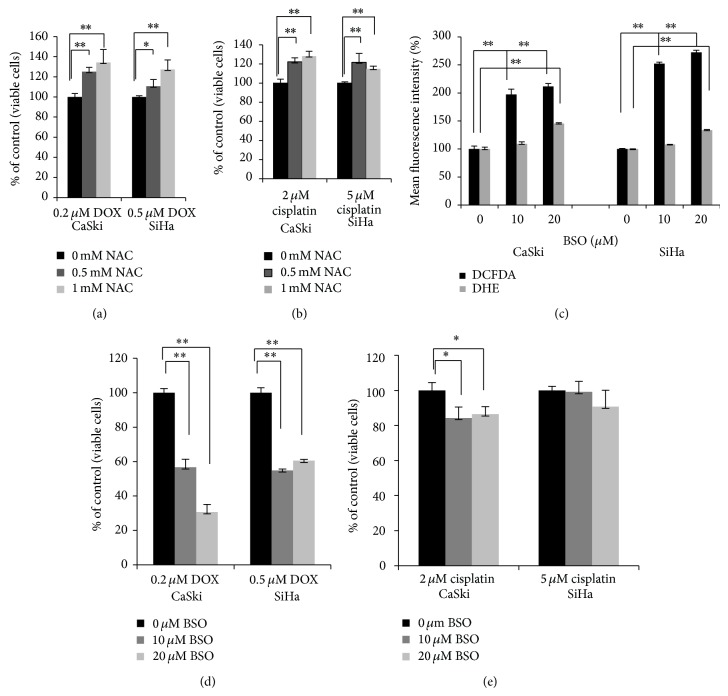
Pretreatment of CaSki and SiHa cells with NAC decreases oxidative stress and protects them from death induced by DOX (a) and cisplatin (b), while pretreatment with BSO increases ROS (c) and sensitizes cells to cell death induced by DOX (d) and cisplatin (e). (a and b) SiHa and CaSki cells (1 × 10^4^ cells per well) were seeded into 96-well plate and then pretreated with the indicated concentrations of NAC for 4 h followed by treatment with DOX for 48 h (a) or cisplatin for 48 h (b). The viability of cells untreated with drugs in the presence or absence of NAC cells was set at 100%. (c) 10^5^ CaSki or SiHa cells were treated with 10 or 20 *μ*M BSO for 24 h and ROS measurements were performed as described in Figure 3(a). (d and e) Cells (1 × 10^4^ cells per well) were seeded onto a 96-well plate and then pretreated with indicated concentrations of BSO for 24 h followed by treatment with DOX (d) or cisplatin (e) for 48 h. Viability was assessed by crystal violet staining. The viability of cells untreated with drugs in the presence or absence of BSO was set at 100%.

We next asked whether a change in ROS levels would also affect the cellular response to actual chemotherapeutic drugs. Cells were pretreated with NAC and then exposed to DOX (Figure 4(a)) or to cisplatin (Figure 4(b)). Pretreatment with NAC for 4 h protected both CaSki and SiHa cells from cell death induced by DOX and cisplatin.

We also asked whether increasing ROS would affect the cellular response to chemotherapy agents by pretreating CaSki and SiHa cells with BSO, an inhibitor of glutathione [15]. Flow cytometry (Figure 4(c)) demonstrated that the ability of BSO to reduce glutathione levels did indeed lead to an increase in cellular ROS as detected by DCFDA and DHE. Pretreatment with BSO sensitized both CaSki and SiHa cells to DOX and to cisplatin (Figures 4(d) and 4(e)), with a greater effect seen with DOX than with cisplatin, and on CaSki cells with their higher basal level of ROS than on SiHa cells. Together, these experiments demonstrate that manipulating the level of cellular oxidative stress can modify the cellular response to chemotherapy agents, such that higher levels of ROS level sensitize cells to chemotherapy-induced cell death.

### 3.5. C33A and HeLa Cells Display Differential Sensitivities to DOX and Cisplatin

**Figure 5:**
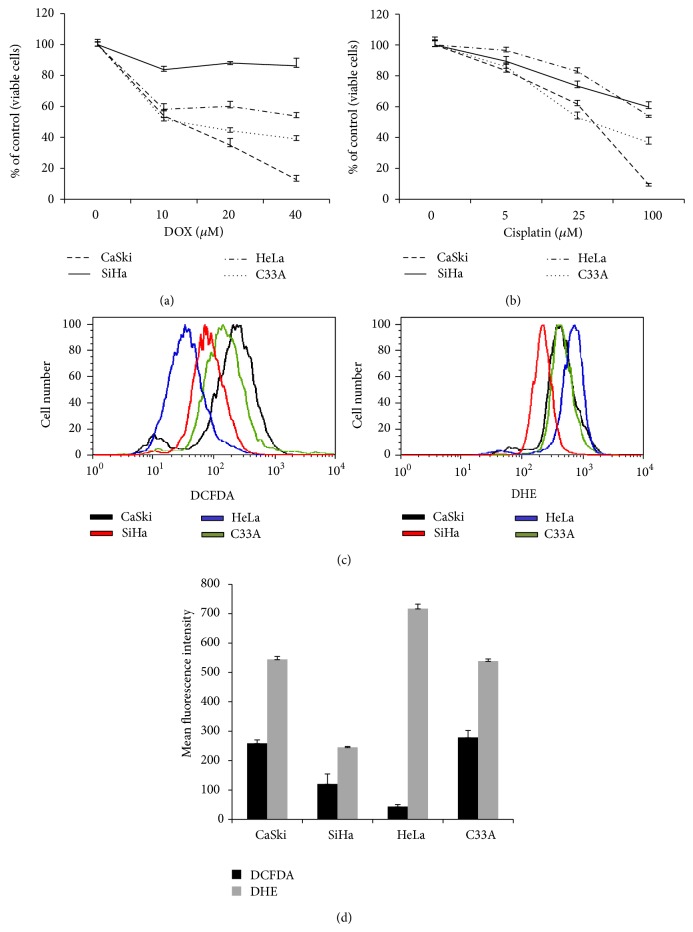
CaSki, SiHa, HeLa, and C33A cervical cancer cells display differential responses to treatment with DOX (a) and cisplatin (b), as well as different baseline levels and distributions of ROS (c and d). (a and b) SiHa, CaSki, HeLa, and C33A cells (1 × 10^4^ cells per well) were seeded into a 96-well plate, allowed to incubate overnight, and then treated with the indicated concentrations of DOX (a) or cisplatin (b) for 48 h. Viability was measured by crystal violet staining and the viability of untreated cells was set at 100%. Each measurement was done in triplicate and error bars indicate the standard deviations of the means. (c and d) 10^5^ cells of each line was treated with 10 *μ*M DCFDA or 10 *μ*M DHE in media and then incubated in the dark at 37°C for 30 minutes. The cells were then washed, and following trypsinization were resuspended in 1x PBS and were analysed by flow cytometry. A total of 10 000 events were measured per sample. (c) DCFDA was detected in the FL-1 channel, while DHE was detected in the FL-2 channel. (d) Bar graphs show triplicate measurements of mean fluorescence intensity of DCFDA or DHE in SiHa, CaSki, HeLa, and C33A cells. Error bars represent the standard deviation.

The results described above demonstrate that higher intracellular levels of ROS increase sensitivity to DOX and cisplatin in the CaSki and SiHa cervical carcinoma cell lines. To further explore the connection between ROS levels and the cellular response to such agents, two additional cervical cancer cell lines, HeLa and C33A, were analyzed for their response to DOX and cisplatin as well as for their intercellular levels of ROS. Cell viability following treatment with these agents is presented in Figures 5(a) and 5(b). Interestingly, of these four tested cell lines, CaSki cells remain the most sensitive and SiHa the most resistant to both agents. C33A and HeLa cells displayed an intermediate sensitivity, with C33A cells more sensitive than the HeLa cells to both agents. Interestingly, the resistance displayed by HeLa cells to cisplatin treatment was very similar to that seen in SiHa cells (Figure 5(b)), although HeLa cells were more sensitive to DOX than were SiHa cells (Figure 5(a)).

Differences in the level of ROS as measured by DCFDA and DHE between these four cell lines were also noted (Figures 5(c) and 5(d)). In particular, while C33A cells displayed intermediate levels of ROS as assessed by both DCFDA and DHE, HeLa cells displayed the lowest levels of ROS as detected by DCFDA and the highest levels of ROS as detected by DHE.

### 3.6. DOX and Cisplatin Treatments Induce Different ROS Profiles

**Figure 6:**
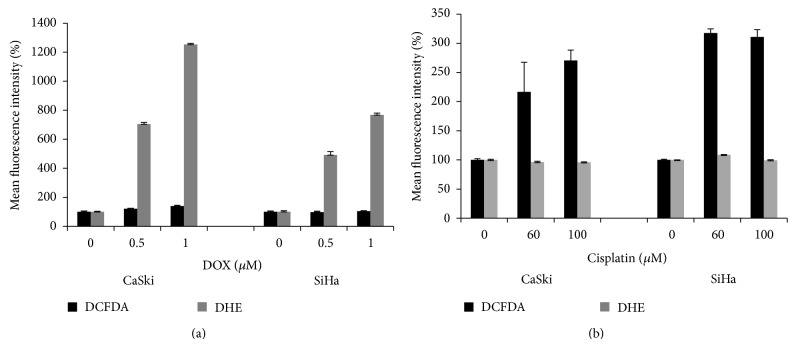
Treatment of CaSki and SiHa cells with DOX increases the level of ROS detected by DHE (a), while treatment with cisplatin increases the level of ROS detected by DCFDA (b). 10^5^ CaSki or SiHa cells were treated with 0.5 or 1 *μ*M DOX for 2 h (a) or with 60 or 100 *μ*M cisplatin for 4 h (b). DCFDA or DHE was added to the media to a final concentration of 10 *μ*M and cells were incubated in the dark at 37°C for 30 minutes. Cells were then washed, resuspended in 1x PBS, and analysed using flow cytometry. A total of 10 000 events were measured per sample. DCFDA was detected in the FL-1 channel, while DHE was detected in the FL-2 channel. Bar graphs show triplicate measurements of the mean fluorescence intensity of DCFDA or DHE in SiHa and CaSki cells, expressed as 100% of the value observed in untreated cells.

One question raised by the previous experiments concerned the differential response of HeLa cells to treatment with DOX (intermediate sensitivity, similar to that seen with the C33A cells)* versus* treatment with cisplatin (quite resistant, similar to that seen with SiHa cells). The mechanisms by which these two agents induce cytotoxicity differ significantly; cisplatin cross-links the DNA, while DOX intercalates into the DNA and produces DNA lesions. This difference in mechanism suggested that these two agents likely triggered different cell death pathways, with different effects on oxidative stress. To ask if this were indeed the case, CaSki and SiHa cells were treated with DOX or cisplatin and changes in ROS levels were assessed by flow cytometry. The results (Figure 6) showed, somewhat unexpectedly, that DOX and cisplatin induced different profiles of ROS. Cisplatin treatment, in both cell lines, primarily increased the levels of agents detected by DCFDA (H_2_O_2_, hydroxyl and peroxyl radicals (Figure 6(b)), while DOX treatment increased primarily the levels of superoxides as detected by DHE (Figure 6(a)). A similar effect, showing DOX-induced increases in superoxide (DHE) and cisplatin-induced increase in hydrogen peroxide, hydroxyl radicals, and peroxyl radicals (DCFDA), was noted in HeLa and C33A cells (data not shown).

**Figure 7:**
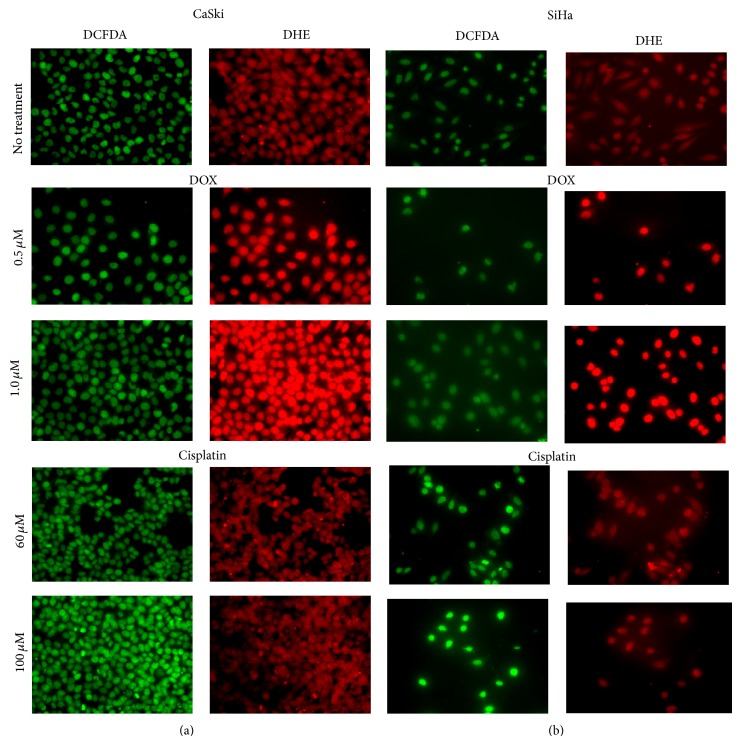
Microphotograph of CaSki (left two panels) and SiHa (right two panels) cells treated with DOX and cisplatin.

To visualize the difference in the levels of ROS radicals detected by DCFDA and DHE staining after DOX or cisplatin treatment of CaSki and SiHa cells, microscopic analysis was performed. Fluorescent images on microphotographs (Figure 7) confirmed the differences previously detected by flow cytometry, showing that (1) overall levels of ROS are higher in CaSki than in SiHa cells; (2) treatment with DOX preferentially increases radicals detected by DHE; and (3) treatment with cisplatin preferentially increases radicals detected by DCFDA.

Together, these results demonstrate that the cellular response to chemotherapeutic agents depends not only on the overall or total levels of ROS, but also on levels of specific oxygen species as well as the mechanism through which these agents exert cytotoxicity.

## 4. Discussion

Resistance to anticancer agents is a major concern in the treatment of cervical and other cancers. Frequently, this resistance appears to be due to alterations in the activation of survival pathways that enable escape from treatment-induced cell death. Identification of these events has the potential to identify new therapeutic targets and sets of biomarkers that could guide clinicians in their selection of treatment options.

In this report, we asked which pathways had the potential to contribute to the drug resistance observed for some cervical cancers. As our initial model, we selected two cell lines, CaSki and SiHa, as representatives of invasive cervical carcinoma. Although both cell lines result from HPV-mediated transformation, the two lines respond quite differently to treatments with chemotherapeutic drugs (Figure 1). Proteomic analysis allowed us to identify several pathways with the potential to impact these differential responses (Figure 2(a)). First, we were able to detect a 2.5-fold difference in the level of p53 between SiHa and CaSki cells. However, since the absolute levels of p53 are low in both cell lines due to accelerated degradation of p53 by HPV16 E6 [12], this cannot be the only contributor to the observed differential drug resistance. Likewise, the presence of mutant p53 in C33A cells [16] means that the p53 response is unlikely to contribute significantly to the C33A response to treatment. Other differences identified by our proteomic analysis pointed toward proteins involved in mitochondrial functioning, as these have the potential to influence the production of free radicals and ROS homeostasis. PCR analysis of an array of genes involved in ROS regulation confirmed differences in the expression levels of several of these genes and also identified differences in the expression of additional genes involved in the regulation of ROS (Table 1). These findings are consistent with the previous observation that, in the absence of p53, ROS itself may act to signal DOX-induced cell death [17].

The major source of ROS production in cells is the mitochondria, where enzymes involved in the electron transport chain and the production of superoxide are located [18]. ROS-producing enzymes identified in the present study were expressed at higher levels in CaSki cells (Table 1). On the other hand, the expression of proteins with antioxidant functions was higher in SiHa than in CaSki cells. Examples of such antioxidant enzymes include SOD1, SOD2, NQO1, PRDX, and GPX (Figure 2(b), Table 1 and Table 1, Supplementary Material). These differences have downstream consequences, as we observed that the differences between SiHa and CaSki cells in the expression levels of proteins involved in ROS metabolism were reflected in the cellular levels of ROS as measured by flow cytometry (Figure 3(a)). Furthermore, these differences were also reflected in the difference in levels of expression of proteins involved in DNA damage recognition and response (Table 1, Supplementary Material), since these processes are activated during oxidative stress [19, 20].

The connection between chemoresistance and a high level of antioxidant defense has been shown previously for other types of cancer, especially for those in advanced stages. An upregulated antioxidant capacity not only allows cells to survive under conditions of oxidative stress, but also provides a mechanism for adapting to exogenous stresses such as treatment with anticancer agents. For example, resistance to arsenic trioxide by bladder urothelial carcinoma cell lines [21] and myeloma cells [22] was found to be associated with an upregulation of heme oxygenase (decycling)1, SOD1, and glutathione reductase. Also, resistance to agents that induce intracellular ROS production, such as paclitaxel, DOX, or platinum-based drugs, is correlated with increased antioxidant capacity in hepatoma cells [23, 24]. One of the most important antioxidant enzymes is SOD2, also known as MnSOD, which catalyzes the conversion of superoxide radicals to H_2_O_2_. SOD2 is also considered to function as a negative modulator of cellular apoptosis and as a prosurvival factor for cancer cells [25].

The majority of anticancer drugs in clinical use are thought to act primarily by way of DNA damage or microtubule disruption. For example, cisplatin and mitomycin C are DNA-damaging agents that form bifunctional DNA adducts, leading to activation of the cellular response to DNA-damage or to DNA damage-induced apoptosis. However, cisplatin may also be able to induce apoptosis in the absence of nuclear DNA [26], and cisplatin-induced cell death in renal cortical cells was shown to involve peroxidation and the release of calcium from intracellular stores [27]. Based on these and other studies, the antitumor effect of cisplatin is now considered to be due to a combination of nuclear and nonnuclear effects including ROS induction, peroxidation, and lethal cell injury [28, 29]. Anthracyclines, such as DOX, are classified as inhibitors of topoisomerase-II; however, the toxic side effects of doxorubicin have long been known to involve oxidative events as well. In fact, oxidative damage is now considered an important factor in the antitumor activity of DOX [30]. In fact, strategies designed to manipulate levels of ROS are now considered a major focus of cancer chemotherapy [31, 32].

We found that treatment with both DOX and cisplatin led to increased levels of ROS (Figures 6 and 7). Unexpectedly, however, we found that the two agents induced quite different profiles of these reactive oxygen species (Figures 6 and 7). DOX increased the DHE signal (superoxides), but did not change the levels of those species detected by DCFDA (H_2_O_2_, hydroxyl, and peroxyl radicals) (Figures 6(a) and 7). In contrast, cisplatin considerably increased the DCFDA signal, but did not significantly increase the DHE signal (Figures 6(b) and 7). Consistent with these findings, previous work had found that treatment with DOX increased the level of superoxides, but not of H_2_O_2_, hydroxyl, or peroxyl radicals in HaCaT keratinocytes [33, 34]. In contrast, cisplatin primarily increased the level of hydroxyl radicals but not of superoxides in human hair follicle dermal papilla cells and in HaCaT keratinocytes [35].

The mechanism of ROS generation induced by DOX is controversial and not yet fully understood [36, 37]. It is known that in the presence of molecular oxygen, the derivatives from “redox cycle” are acted upon by a number of NAD(P)H-oxidoreductases cytochrome P450 or cytochrome-*b*5 reductases, mitochondrial NADH dehydrogenase, xanthine dehydrogenase, endothelial nitric oxide synthase (reductase domain) to generate superoxides [38–40]. One-electron “redox cycling” of DOX also produces superoxide; this process is accompanied by the release of iron from intracellular stores and results in formation of drug-iron complexes that release superoxides and hydrogen peroxides [41], which can then be decomposed by antioxidant systems. The higher levels of superoxide observed during DOX treatment are believed to be due to a decrease in the activity of superoxide-decomposing enzymes such as MnSOD and catalase [34].

ROS generation by platinum-based drugs is also not fully understood. The predominant formation of hydroxyl radicals is believed to be the result of peroxynitrite decomposition in cisplatin-treated cells [35]. After penetration into the cells, alkylating agents such as cisplatin bind to glutathione, and this interaction leads to removal of this complex from cells through an ATP-binding cassette. Depletion of glutathione then results in increased H_2_O_2_ and hydroxyl radicals [35, 42]. The difference in the mechanism of action between DOX and cisplatin is supported by experiments in which cells were sensitized by BSO pretreatment prior to exposure to the drugs (Figures 4(d) and 4(e)). The lower level of sensitization to cisplatin can be explained on the basis that BSO and cisplatin act on the same substrate, glutathione. Therefore, we speculate that glutathione depletion as a result of BSO treatment does not change cell viability dramatically after administration of cisplatin, because the substrate for cisplatin toxicity is already depleted.

To examine the dependence of the cellular response to therapeutic agents on oxidative stress, we manipulated ROS levels by either depleting ROS through pretreatment with NAC or increasing ROS through pretreatment with BSO. Pretreatment with NAC or BSO, reducing or increasing, respectively, the levels of ROS (Figures 3(c) and 4(c)) were able to protect or to sensitize cells to both agents, respectively (Figures 4(a), 4(b), 4(d), and 4(e)). We also demonstrated that cells with higher baseline levels of ROS experienced a more rapid loss of viability following drug treatment than did cells with lower baseline levels of ROS. In particular, CaSki and C33A cells, which display higher levels of ROS, died faster than did SiHa cells following treatment with either DOX or with cisplatin (Figures 1 and 5). In the case of Hela cells, however, the cellular response to DOX differed from the response induced by cisplatin (Figures 5(a) and 5(b)). This may be due to the different profiles of reactive oxygen species induced by the two agents (Figure 6). Based on these data, a preferred chemotherapy for tumors exhibiting a ROS profile similar to that of HeLa cells, for example, might favor DOX over cisplatin. Our experimental results demonstrated that different cervical cancer cell lines differ not only in their total level of ROS, but also in the levels of specific free radicals. Ideally, clinical selection of chemotherapeutic treatments should consider the types of ROS induced, which will be related to the mechanism of drug action at the molecular level.

The data in this report provides evidence that differences in the sensitivity of cervical cancers to chemotherapeutic treatments is likely due, at least in part, to differences in the relative levels of pro- and antioxidant enzymatic activity that lead to different baseline levels of oxidative stress. Chemoresistance is undoubtedly a multifactoral process and factors in addition to high levels of antioxidant defense are likely to contribute. Such factors may include differences in angiogenesis, which could affect the penetration of agents into tumor tissue, increased drug efflux from the cancer cells, reduced uptake of drugs, interactions of cancer cells with their surrounding microenvironment, and other factors [43–45]. Such factors are also likely to contribute to the chemoresistance of cervical carcinomas. A better understanding of how and when these various factors, including the baseline levels of oxidative stress, affect how a particular tumor will respond to a specific treatment has the potential to improve our treatment of patients suffering from cervical malignancies.

## 5. Conclusions

We utilized a proteomic approach to identify the pathways involved in resistance to the chemotherapeutic agents cisplatin and doxorubicin and then extended these findings by analyzing and comparing the expression level of genes involved in reactive oxygen species (ROS) metabolism through the use of an qRT-PCR array. These data enabled us to demonstrate that pathways involved in oxidative stress and antioxidant defense contribute to drug resistance. In particular, the sensitive CaSki cells expressed lower levels of antioxidant enzymes, resulting in higher levels of ROS, than did the resistant SiHa cells. Decreasing or increasing oxidative stress using pharmacological agents led to protection or sensitization, respectively, in both cell lines, supporting the idea that cellular levels of oxidative stress affect responsiveness to treatment. Interestingly, the two agents tested, doxorubicin (DOX) and cisplatin, induced different profiles of ROS, and these differences appear to contribute to the differential sensitivity displayed by cervical cancer cells to treatment.

## Supplementary Material

“Table 1 of the Supplemental Material lists the 430 genes for which the level of expression between SiHa and CaSki cells differed by more than 1.5 fold.” Also, we included a description of this data underneath the heading “Supplementary Material” in the text.

## Figures and Tables

**Table 1:** Relative expression (SiHa *versus* CaSki) of genes involved in oxidative stress and antioxidant defense.

	Genes	Fold	St. dev.
Downregulated genes	AOX1	−3.91951	2.099233
	NCF2	−73.405	14.30477
	OXR1	−3.49989	0.529737

Upregulated genes	CYBA	4.275	2.269813
	DUSP1	6.58	2.870854
	GPX1	1.6	0.381838
	GPX5	2.085	1.421285
	GPX6	2.625	2.043539
	GSS	2.085	1.421285
	PRDX1	4.315	0.982878
	PRDX2	13.195	11.10865
	SELS	5.635	1.916259
	SOD1	7.745	4.249712
	SOD2	3.84	0.127279
	SOD3	3.055	2.38295
